# Tactile regulation with pocket-sized robots: reducing movement and heart rate in children through hand-held interaction

**DOI:** 10.3389/frobt.2026.1810778

**Published:** 2026-09-23

**Authors:** Morten Roed Frederiksen, Kasper Støy, Maja Matarić

**Affiliations:** 1 Data Systems and Robotics, IT-University of Copenhagen, Copenhagen, Denmark; 2 Interaction Lab, University of Southern California, Los Angeles, CA, United States

**Keywords:** behavioral interventions, embodied interaction, heart rate, movement regulation, pocket-sized tactile devices, restlessness, robotics, tactile interaction

## Abstract

Periods of heightened arousal or restlessness can interfere with children’s ability to remain physically calm in everyday settings such as classrooms. Handheld tactile technologies may offer a simple and engaging way to support brief moments of structured sensory-motor engagement. This paper investigates how a pocket-sized tactile device influences physiological and behavioral markers of calmness in typically developing children. Building on prior work examining heart-rate modulation, we present new findings on how a handheld rhythm-matching interaction affects full-body movement and postural stability. Eighteen children participated in a within-subjects study involving two conditions: active interaction with a handheld tactile rhythm-matching game and a comparison condition in which they held the same device with the tactile interaction disabled. Results showed lower heart rate during active interaction, with an average reduction of 3.37 bpm (p = 0.019), and lower overall body movement, with movement decreasing by 37.6% (p = 0.002). Regional movement analyses showed the largest reductions in head, neck, and shoulder movement (44.8% reduction) and upper-limb movement (48.5% reduction). These findings suggest that brief tactile rhythm-matching interaction with a handheld device is associated with reduced acute physiological arousal and physical restlessness in children. The study provides a preliminary baseline for future work examining how tactile robotic interactions may support calmness in more demanding or stressful contexts.

## 1 Introduction

Supporting children’s ability to self-regulate, control impulses, and be able to be physically calm is central to healthy cognitive and emotional development (Blair and Diamond, 2008; Nilfyr and Ewe, 2025). In everyday contexts such as classrooms and home environments, children experience states of heightened arousal, restlessness, or difficulty maintaining focused attention (Ciucci et al., 2016; Nkrumah et al., 2015). These conditions can manifest through physiological indicators such as elevated heart rate, and behavioral expressions such as fidgeting or excessive movement (Komissarova and Fomina, 2023; Dogadkina et al., 2022; Bang et al., 2017). Finding accessible and engaging ways to help children regulate these responses remains an ongoing challenge.

Traditional approaches to fostering calmness in children, such as mindfulness exercises and breathing techniques, can be effective but often require repeated instruction, structured practice, and adult facilitation (Li et al., 2021; Jain, 2021).

As a complement to such methods, interactive technologies that provide immediate or delayed sensory feedback may offer a simple and playful pathway toward increased attention and self-regulation (Ayearst et al., 2023; Basu et al., 2022). Tactile engagement, in particular, can redirect attention to the body and promote stillness through rhythmic or patterned touch (Bincy and John, 2019; Davies et al., 2019). Intelligent technology has the potential to address key gaps in existing interventions by delivering immediate forms of support that require little or no prior training, potentially making it more accessible to self-regulate emotions in difficult scenarios (Rasouli et al., 2022). This research explores whether a small hand-held tactile device can influence physiological and behavioral markers of calmness in typically developing children. Building on our earlier study showing reductions in heart rate during tactile interaction with a pocket device, here we extend the analysis to include movement tracking to assess changes in general restlessness and postural stability (Frederiksen et al., 2024a). Rather than testing clinical efficacy or long-term self-regulation abilities, the present study examines whether a brief rhythm-based tactile interaction is associated with lower heart rate and reduced movement compared with a similar interaction with an inactive device. By investigating acute physiological and movement responses in a typically developing child population, we establish a preliminary baseline for future studies of tactile interactions in more demanding or stressful contexts.

### 1.1 Tactile interaction, self-regulation, and movement

Previous research in child–technology interaction has shown that gentle touch, rhythmic feedback, and simple embodied tasks can influence attention and emotion regulation (Bincy and John, 2019; Ren and Belpaeme, 2025). Tactile engagement provides a form of sensory grounding that may assist children in maintaining focus and reducing unnecessary movement (Cha, 2023). Additionally, related work in sensory integration and self-regulation has demonstrated that tactile input can stabilize posture and modulate physiological states by guiding awareness toward the body (Frederiksen et al., 2024a; Wang et al., 2021; Sabinson et al., 2021). Similarly, socially assistive and therapeutic robots have shown promise as means of encouraging calmness, focus, and engagement through haptic or rhythmic cues (Kimura and Tanaka, 2023; Sefidgar et al., 2016; Ma et al., 2021).

Electrodermal activity (EDA) is also widely used as a marker of sympathetic arousal and would be a relevant physiological measure in future studies (Roy et al., 2012; Critchley, 2002). However, EDA was not included in the present study because our protocol intentionally allowed children to move naturally, and wearable EDA recordings can be sensitive to motion artifacts, electrode contact, and ambulatory measurement conditions (Tronstad et al., 2022; Posada-Quintero and Chon, 2020; Hossain et al., 2022). While much of prior research on self-regulation has focused on internal physiological responses such as heart rate or breathing, children’s ability to remain calm also depends on external behavioral control (Nilfyr and Ewe, 2025; Eisenberg et al., 2010). Small, seemingly spontaneous body movements, such as fidgeting, shifting weight, or moving the arms and legs, often reflect fluctuating levels of arousal (Rosenbaum et al., 2021; Gerdemann et al., 2025). Importantly, the functional meaning of fidgeting is context-dependent. Fidgeting and purposeful movement are sometimes interpreted as signs of restlessness, but they may also serve regulatory functions, such as helping maintain arousal, reduce discomfort, or support engagement during tasks. We therefore interpret movement reduction as an acute behavioral change during tactile interaction, rather than as direct evidence of improved attention or self-regulation. These movements can interfere with attention and signal reduced calmness (Raghunathan et al., 2022).

In this study, we investigated whether tactile interaction with a pocket-sized robot is associated with reduced body movement. We frame the interaction as a structured sensory-motor anchor: the child holds the device, attends to rhythmic tactile cues, and responds through simple button presses. This framing allows us to examine whether a brief tactile rhythm-matching task is associated with lower movement and heart rate, without assuming that the specific psychological mechanism is already established. The research extends our previous work (Frederiksen et al., 2024a) that reported findings on heart rate reduction during interaction with a pocket-sized tactile device. While the earlier study focused exclusively on physiological heart rate indicators, this work presents novel findings about physical movement patterns in the same experimental context. Our previous work established that interactions with our tactile robot significantly reduced heart rate (p=.019) by approximately 3–5 bpm compared to non-interaction conditions Frederiksen et al. (2024a). In this work, by examining physiological and behavioral dimensions together, we provide a more complete understanding of the potential of using tactile interactions to influence embodied self-regulation in children.

This work addresses the following research questions.

- RQ1: How does tactile interaction with a pocket-sized device influence physical markers of elevated arousal in children compared with holding the inactive device?
- RQ2: How does interaction with a pocket-sized tactile robot in a rhythm-matching task influence children’s heart rate compared with holding the inactive device?
- RQ3: What is the relationship between heart-rate change and movement change during tactile interaction with a pocket-sized device?

Based on these research questions, we formulated four hypotheses.

- H1: Children’s total physical movement will be significantly reduced during active engagement with the robot’s tactile game compared with the Without-Interaction condition.
- H2: The children’s upper body parts (head, eyes, and upper body) will show greater movement reduction than other body areas during tactile interaction with the pocket-sized robot.
- H3: The magnitude of the children’s heart rate reduction will positively correlate with the degree of movement reduction.
- H4: The magnitude of the condition difference will be time-dependent, resulting in a more pronounced difference between the With-Interaction and Without-Interaction conditions during the final phase (the last 60 s) of the session compared to the initial phase.

To answer these questions, we designed and built a simple, low-cost pocket-sized device that offers tactile vibratory interactions. By focusing on repeated button-press sequences, reinforced through gentle vibration, the device is designed to hold the user’s attention during interaction (Frederiksen et al., 2024a). Through a 14-day pilot study and a subsequent three-day in-school user study (combined n=20 participants), we gathered data from children interacting with the device, including heart-rate and video-derived joint movement tracking over time.

Our work examines these mechanisms using a pocket-sized tactile device (shown in Figure 1) that invites the user to engage in rhythmic matching through vibration and button presses. As a small form factor ubiquitous technology in the shape of a small robot, the device is conceptualized as a tool for embodied self-regulation. It is developed to explore how tactile feedback and attention coupling can influence physiological calmness. By studying changes in users’ heart rate and physical movement, we aim to understand how short-term tactile engagement with the device can promote a calmer, more focused bodily state in children.

**FIGURE 1:**
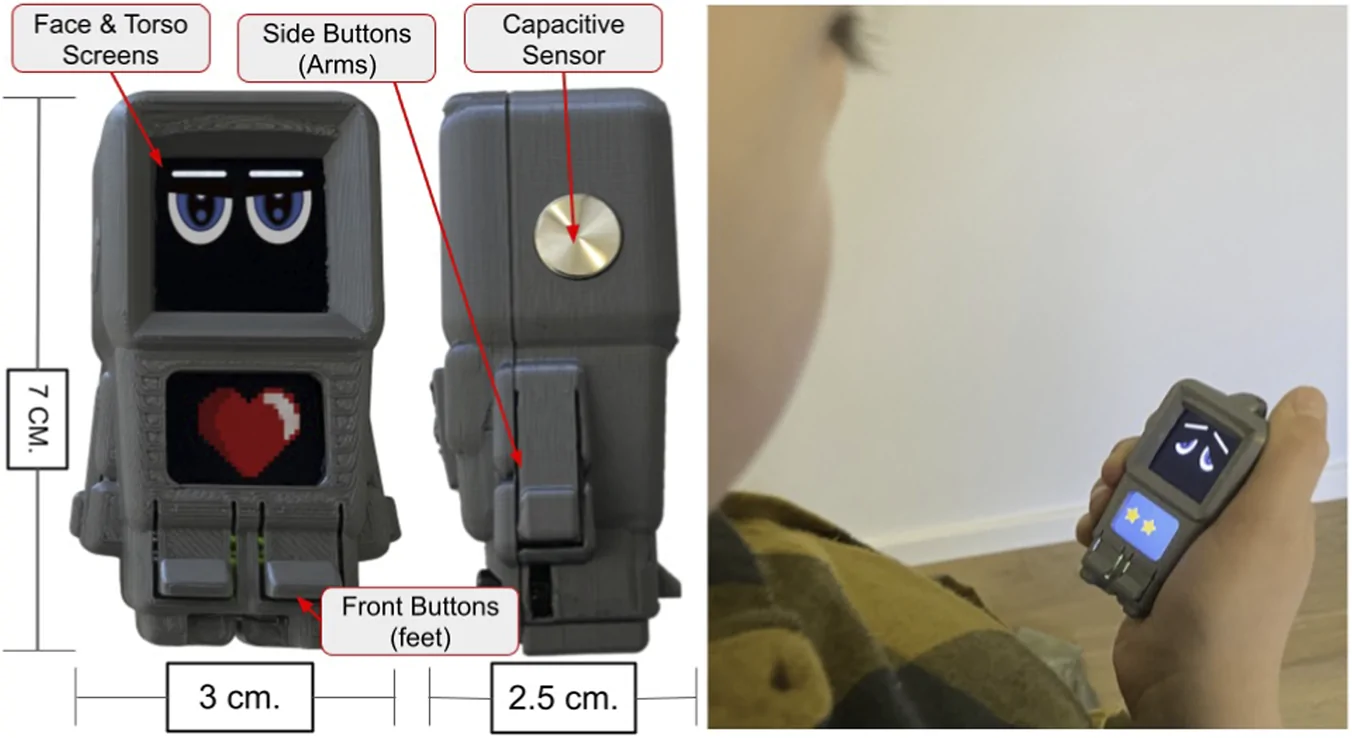
The “AffectaPocket” device, developed to promote physical and emotional self-regulation for children aged 6–12. AffectaPocket is controlled by an onboard EPS32 chip, and powered by a 3.7 V battery. Children interact with the robot through a tactile rhythm-matching game played by grasping the robot in one hand and repeating generated rhythmic patterns.

### 1.2 Behavioral interventions for children

Current therapeutic approaches that promote calmness include a variety of meditative practices. Targeted relaxation training has been shown to decrease motor restlessness and impulsive movement in children, fostering greater physical stability and attention span (Schilling and Poppen, 1983). Research into structured respiration practices demonstrated that brief, daily interventions can effectively reduce physiological arousal (Balban et al., 2023). Self-regulation strategies like self-talk, deep breathing, progressive muscle relaxation, and imagery have been established to reduce physiological arousal. In the physical domain, structured exercise protocols have been shown to have a positive effect on physical arousal (Servant, 2019), with longitudinal research demonstrating improved outcomes from developmentally appropriate physical activity (Melnyk et al., 2009). There is an extensive body of research examining the nexus between bodily focus and psychological wellbeing in pediatric populations. Recent evidence suggests that physiological regulation serves as a critical entry point for cognitive improvement Wang et al. (2024). For instance, digital breath-focused mindfulness training in parent-child dyads has been shown to reduce default mode network activity and sharpen attentional focus in children Jaiswal et al. (2025). Similarly, physical relaxation techniques have proven effective across diverse developmental stages and environments. Progressive muscle relaxation and similar protocols have yielded significant gains in attention and executive functioning for both kindergarten-aged children (5–6 years) and those in institutional settings Jarraya et al. (2022), Nair et al. (2024). Building upon these somatic foundations, recent adaptations of Mindfulness-Based Cognitive Therapy (MBCT) have pivoted toward targeting emotional regulation directly Chambers et al. (2009), Gross (1998), Kuyken et al. (2022).

While traditional mindfulness and physical activity interventions effectively promote calmness, they typically rely on adult facilitation, structured environments, and repeated training. There remains a significant need for immediate, accessible tools that allow children to engage in self-regulation autonomously within everyday contexts. This study addresses this gap by using a minimalist, pocket-sized tactile robot to evaluate whether rhythmic haptic interaction is associated with short-term changes in children’s heart rate and body movement.

### 1.3 Tactile interactions and movement analysis

Beyond physiological indicators, physical movements such as fidgeting, shifting posture, and restlessness can provide observable markers of children’s behavioral state during interaction (Association, 2022; Rapee et al., 2009). Developmental research links self-regulation and attentional control to children’s ability to maintain task engagement and manage behavior (Diamond, 2020; Blair and Diamond, 2008). Prior work on mindfulness, relaxing touch, and robot-assisted mindfulness suggests that structured embodied activities can influence physical movement patterns and physiological state (Thompson and Gauntlett-Gilbert, 2008; Alimardani et al., 2020). Previous research has indicated that engagement with structured tactile tasks produced an “anchoring” effect that reduced autonomic arousal and motor restlessness during game-like sessions (Mary et al., 2001).

While therapeutic studies rarely explicitly focus on movement, some research in the context of autism spectrum disorders has used motion-sensing technologies to correlate physical posture with emotional engagement (Clabaugh et al., 2019; Anzalone et al., 2015). Various studies have also used user pose-tracking to document both engagement and posture changes to provide objective measures of attention, comfort, and interaction quality in therapeutic robotics (Jain et al., 2020; Kennedy et al., 2015). Movement vector analysis, which tracks instantaneous body displacement, is a method that offers quantitative insights into the relationship between movement and emotion (Lang et al., 2000), an aspect that was traditionally measured using wearable devices such as heart rate and EEG monitors (Michels et al., 2013; Mohammadi et al., 2017). Recent research efforts highlight that while socially assistive robots are widely used for motivation, a critical need remains for structured tools to specifically measure the tactile dimension of these therapeutic interactions (Pirborj et al., 2025; Arnold and Scheutz, 2017; Paterson, 2022). Parallel to this, the development of sensor-integrated fidget objects has enabled the objective quantification of restlessness, validating fidgeting patterns as measurable data points for monitoring health states (Bobrova and Perego, 2025; Bobrova et al., 2024; Bobrova, 2025). Physiological regulation has also been achieved through ‘affective stroking’ using mid-air haptics, where simulated tactile sensations successfully shifted autonomic states toward relaxation (He et al., 2024). Furthermore, new methods in sensory substitution demonstrate that the emotional intent of tactile gestures can be accurately recognized through sonification, implying that touch contains extractable data features distinct from physical contact (de Lagarde et al., 2025). These findings align with theoretical frameworks that increasingly view therapeutic touch as a bidirectional communicative act, where improved ‘fluency’ in tactile exchange correlates directly with better functional movement outcomes (Tuttle and Hillier, 2023).

### 1.4 Technical behavioral interventions for children

Almost 30 years of research into the use of robots with socially assistive capabilities has demonstrated a wide range of positive effects, including on physical rehabilitation for children with motor disabilities (Buitrago et al., 2020). Socially assistive robots (SARs) are designed to support users through social interaction rather than physical task execution (Feil-Seifer and Matarić, 2005). In child-facing contexts, SARs have been explored as engaging mediators for interaction, coaching, storytelling, and structured activities (Spitale et al., 2022; Lakatos et al., 2023; Leite et al., 2014).

Therapeutic robots have been deployed in diverse applications to support children, from relaxation-oriented designs like the pillow-shaped TACO robot (O’Brien et al., 2021), and the huggable Probo robot (Saldien et al., 2010) to pet-inspired interactions for pain reduction such as with the Maki robot and the Paro robot seal (Kelly et al., 2021). These robots aim to build lasting relationships (Díaz et al., 2011), and provide relaxing interactions (Rabbitt et al., 2015). Robot companions specifically designed for children can reduce distress and build skills (Arnold, 2016; Jeong et al., 2015). These technologies address challenges by providing opportunities for social skill practice (Zhu and Deng, 2021), while facilitating relaxation techniques such as deep breathing and interventions that train calmness over time (Matheus et al., 2023; Matheus et al., 2022; Shao et al., 2019; Crossman et al., 2018).

Multiple studies document tangible benefits of repeated therapeutic interactions with robots for children with physical or cognitive disabilities (Alemi et al., 2016; Taheri et al., 2017; Marti et al., 2005). In clinical settings, in particular hospitals, socially assistive robots can provide relaxation when integrated into routine care (Jeong et al., 2018; Okita, 2013). Meta-reviews show encouraging preliminary results across different healthcare contexts (Littler et al., 2021; Logan et al., 2019). Robot-assisted strategies employed in behavioral interventions can be diverse and can range from playful companionship to structured coaching (Admoni and Scassellati, 2017; Rasouli et al., 2022).

Socially assistive robots effectively motivate and aid children, but many platforms are large, cost-prohibitive, and focused on social interaction rather than discreet physical regulation. There remains a need for empirical data on how haptic feedback alone, isolated from broader social cues, influences a child’s physiological and behavioral state.

### 1.5 Contributions

The contributions of this research are as follows:

1. We provide a combined analysis of physiological and movement data showing that children exhibited lower heart rate and reduced movement during active tactile rhythm-matching interaction compared with holding the inactive device.
2. We show that active tactile interaction was associated with a significant (t(17) = 3.75, p = 0.002, 95% CI [36.7, 131.3] cm, Cohen’s $d_{z}$ = 0.88) reduction in overall physical movement, with movement reduced by 37.6% compared with the inactive-device condition.
3. We provide insights into how handheld tactile robotic technology may support brief, accessible, low-training interactions associated with reduced physiological arousal and motor restlessness.

Our findings provide an empirical foundation for studying how tactile rhythm-matching interaction may support short-term physiological and behavioral regulation in everyday settings. The presented study involved children without clinical diagnoses; however, the results may inform future explorations of tactile technologies in broader contexts involving stress, restlessness, or heightened arousal.

## 2 Materials and methods

### 2.1 Participants

We recruited a total of 20 participants (N = 2 for the pilot study; 2 male, and N = 18 for the main study; 10 female, 8 male) aged 6–8 years from a single first-grade class within the Danish public school system. The participants were classmates and familiar with one another but had no interactions with each other during data collection.

Recruitment was coordinated through the school’s administration, starting with an agreement from the head of the school to recruit from a class of 22 students. From this initial pool, three children were excluded because their parents did not provide written consent, and one child was excluded after declining to provide verbal assent. The study protocol was approved by the Research Ethics Committee at the IT University of Copenhagen. To ensure ethical compliance, parents provided written informed consent prior to the study, and each child gave verbal assent immediately before participating. Participation was strictly voluntary, and no compensation was offered to the children or their families. Our experiment was conducted over three consecutive days, in a designated familiar and quiet classroom at the school, between 9 a.m. and 1 p.m. The first half of the children’s school day was selected as a time of highest levels of alertness and cognitive engagement compared to the post-lunch period Latino et al. (2025), Schröder et al. (2016).

### 2.2 Study design

Our research employed a mixed-methods experimental design with the study consisting of two phases: a 14-day pilot study with two participants, followed by a main study with 18 participants. We used a within-subjects design in which each participant experienced both experimental conditions. The conditions were: With-Interaction Condition: Participants actively engaged with the robot’s tactile rhythm-matching interaction by pressing its buttons in response to perceived vibration patterns. Without-Interaction Condition: Participants held the same robot with the tactile game disabled, so the robot provided no vibration. This was the control condition.

### 2.3 Dependent variables

We measured two primary dependent variables in each experimental condition.

- Heart Rate: Continuous measurement of beats per minute (BPM) using a wrist-worn optical heart rate sensor (Polar Variety Sense), providing a physiological indicator of potential arousal. This Photoplethysmography-based (PPG) device was selected for its balance of accuracy and non-invasiveness, minimizing discomfort for the children during the study. Heart rate variability (HRV) rather than heart rate might be more indicative of stress, but current research suggests that measuring HRV demands extended measurement periods incompatible with our brief intervention paradigm (Michels et al., 2013; Shaffer and Ginsberg, 2017; Malik et al., 1996; Laborde et al., 2017). While optical PPG sensors are typically calibrated using adult data, the findings in this work rely on a within-subjects design where each participant serves as their own baseline. By focusing on the relative difference between the control and experimental conditions for each participant, any potential calibration offsets or absolute measurement inaccuracies are held constant across conditions.
- Movement Vectors: Quantitative assessment of physical movement captured through a camera at 30 frames per second, monitoring displacement between frames as an approximate marker of physical movement and postural activity. Using the open-source motion analysis toolkit OpenPose (a joint-based pose tracking Python library), each video frame was analyzed to identify the child’s approximate joint locations Cao et al. (2021), Simon et al. (2017), Cao et al. (2017), Wei et al. (2016). We manually inspected data for artifacts (partial occlusions, child out of frame). Any identified joints with missing measurements were replaced with the last known joint position in the final dataset.

Together, these measures allowed us to examine both a physiological response, heart rate, and behavioral manifestations, movement, during tactile interaction (Healey and Picard, 2005; Sharma and Gedeon, 2012). We interpret these as acute physiological and motor markers rather than direct measures of attention, self-regulation ability, or clinical improvement. By capturing these variables simultaneously, we could investigate potential relationships between physiological calming and behavioral stabilization during tactile interactions. We also conducted an exploratory regional movement analysis focusing on joints in the head, neck, and shoulder region: eyes, nose, neck, and shoulders. These joints were selected because they capture upper-body and head-position changes during handheld interaction and also based on the hierarchy of social cues. In this hierarchy, head orientation can often be used instead of gaze when the eyes are not visible, while body orientation (shoulders/trunk) acts as a coarse indicator of focus direction Emery (2000), Hietanen (2002). Research confirms that orienting to a social stimulus typically involves a coordinated cascade where the head and torso align to stabilize the visual field Doshi and Trivedi (2012), Langton (2000). Here we isolated these features to distinguish specific orienting behaviors from general motor restlessness.

### 2.4 Handheld tactile robot

AffectaPocket is a pocket-sized handheld device shaped like a robot that was specifically designed for children aged 6–12 to promote emotional and physical self-regulation through tactile interactions. The device can be considered a very simple robot because it has very basic intelligent control, it senses through capacitive touch and clickable buttons, and it actuates through an 8 mm Linear Resonant Actuator (Model C08-005, Precision Microdrives) that provides high-fidelity haptic feedback (vibration and button click sensations) with a rated vibration frequency of 235 Hz and a normalized amplitude of 1.28 G. The device measures 7 cm × 4 cm × 3 cm, sized to fit comfortably in a 6–12 year old child’s hand or pocket. Its anthropomorphic design in the form of a toy robot was chosen to encourage engagement and emotional connection and is based on insights from participatory design sessions with children Frederiksen et al. (2024b). The robot has a 3D-printed gray shell with a structured body that includes articulated “arms” (side buttons) and “feet” (front buttons) that serve as clickable points. Its face consists of a small screen displaying animated eyes, while a second screen on its “torso” shows functional information and visual game cues, including a heart graphic during normal operation. The hardware design includes the following key components (shown in Figure 1): capacitive touch sensors embedded in the robot’s body (one circular sensor visible on the side), a battery compartment integrated into the back portion of the design, front buttons positioned as the robot’s feet, side buttons that function as the robot’s arms, and a USB-C charging port. The ESP-32 development board serves as the robot’s brain, providing computational capabilities and Bluetooth connectivity. The user’s primary interaction with the device involves a tactile rhythm-matching game designed to hold children’s attention during moments of restlessness. The game concept is outlined in Figure 2. When grasped for three seconds, the robot generates a three-pulse vibration pattern that the child attempts to replicate by pressing the robot’s side buttons in the correct rhythm. Successful matching triggers tactile feedback through vibration and the game progresses to a new rhythmic pattern to match. This continues until the grasp on the device is released and it is left untouched for 5 s, at which point the pattern-matching process stops. The rhythm-matching game requires the user to press the side buttons simultaneously as each tactile pulse occurs, requiring the child to focus on sensing, remembering, and repeating the rhythmic pattern. A pulse is considered matched if the button press occurs within 300 m of the pulse onset and duration. The robot repeats the full rhythm pattern, and each pulse only needs to be matched once across all attempts. After three unsuccessful attempts, the timing threshold gradually expands with 10 percent in each direction (early, late), continuing until the user just have to grasp the device to match the rhythm, to ensure eventual success. This design choice aims to prioritize user engagement and accomplishment over challenge, in order to promote positive interaction experiences for children with various ability levels. The robot provides an initial tutorial session where a visual aid helps children to understand the interaction concept; after that, the robot is used without visual cues, allowing for discrete operation when concealed in a pocket. This design choice specifically addresses the need of children to play privately and not draw attention to self-regulating strategies.

**FIGURE 2:**
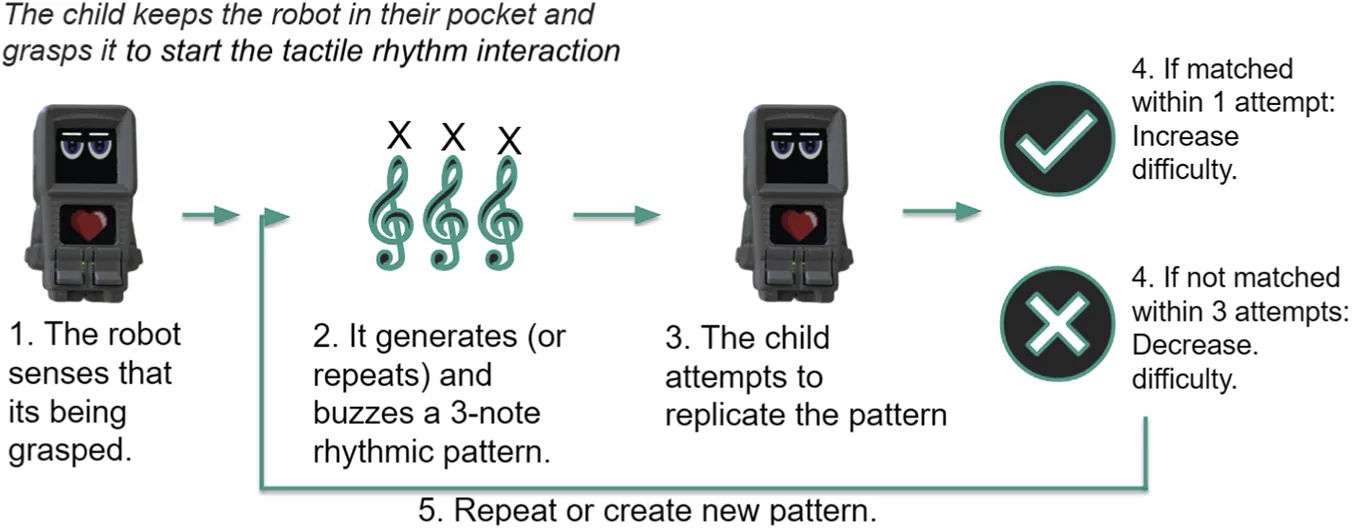
The rhythm matching interaction cycle of the AffectaPocket. The interaction is meant to occur while the robot is concealed in a pocket. The child initiates the interaction by grasping it. The robot proceeds by generating a random 3-pulse rhythm. The child attempts to replicate the rhythm. If the child succeeds in matching all pulses, a new rhythm is generated, slightly harder if the rhythm was matched in first or second attempt. The demands for precision are lowered if 3 attempts has passed with no matched rhythms.

### 2.5 Procedure

The pilot study and main study followed the same core procedure, with participants completing both the With-Interaction and Without-Interaction conditions in randomized order. The procedure was designed to keep the physical setup consistent across conditions, so that the primary difference between conditions was whether the tactile rhythm-matching interaction was active. However, the active condition also required children to perform a structured rhythm-matching motor task, whereas the comparison condition involved passively holding the inactive device. The present comparison should therefore be understood as active tactile rhythm-matching interaction versus passive device holding.

The study procedure was as follows:

1. Each participant was fitted (by the experimenter) with the heart rate tracking band on their upper non-dominant arm.
2. The participant was given a short introduction by the experimenter on how to interact with the robot. This included how to grasp the robot and how to match the rhythm of the vibrational pulses.
3. The participant was asked by the experimenter to stand in the middle of the room, on a spot on the ground marked with red tape.
4. The same room was used for all participants throughout the 2 weeks of the experiment.
5. Heart rate tracking was initiated.
6. The participant then completed both experimental conditions in counterbalanced order:
  - Condition 1. With-Interaction: initiate the tactile interaction game by grasping the robot’s side buttons for three seconds.
  - Condition 2. Without-Interaction: stay within the designated test area for one minute holding the robot without it having the tactile interactions enabled.
7. A timed 20 min break was held in between the two conditions.
8. Each condition lasted 1 min in the pilot study, but was extended to two minutes for the main study.

The experimental setup is illustrated in Figure 3. For both conditions, the child was allowed natural movement (they were not given instructions to stand or keep still in either conditions). This methodological choice was based on research showing that children have limited attention spans (chronological age plus 1 min) and that fidgeting represents a normal self-regulatory mechanism for attention and stress (Farley et al., 2013; Swartz et al., 2020). It is possible that increased fidgeting in the Without-Interaction condition contributed to higher heart rate, since physical activity can elevate heart rate. However, this relationship is also part of the embodied regulation process investigated in this study: if tactile interaction reduces restlessness and unnecessary movement, a corresponding reduction in heart rate may reflect the combined physiological and behavioral calming effect of the interaction. We therefore interpret heart-rate reduction and movement reduction as complementary markers of embodied calmness, while acknowledging that the present design cannot fully separate direct autonomic effects from movement-mediated changes in heart rate.

**FIGURE 3:**
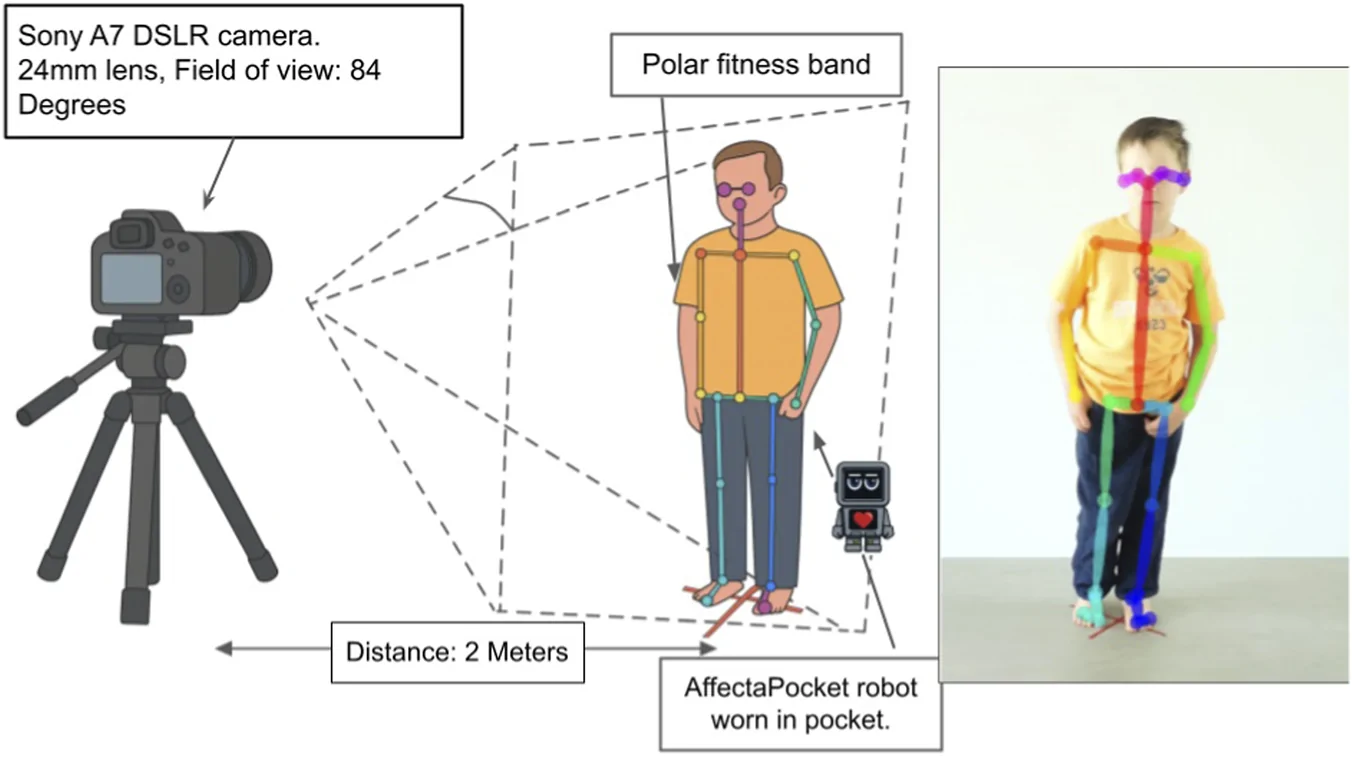
Left: The experimental setup of the main study. The participants were standing 2 m away from the camera with the tactile robot in their pocket. Their heart rate were measured by a Polar heart rate tracker placed at the upper part of their dominant arm. Right: A participant interacts with the AffectaPocket in their pocket. The coloured lines are the tracked joints outputted by the open source joint pose tracking library.

### 2.6 Pilot study: initial heart rate assessment

We conducted a preliminary 14-day pilot study with two typically developing children (aged 8 years) to evaluate AffectaPocket’s impact on heart rate and to validate the above outlined study procedure. Testing was conducted in participants’ homes at consistent times (3–4 pm daily), with each interaction lasting one minute. Participants completed the two study conditions daily, in randomized order (determined by a coin toss), separated by a 20-min wash-out interval during which the participants left the device on a nearby table. During the study conditions, the robot remained in participants’ pockets. The participants were free to choose which pocket and which hand they used when interacting with the device. They put their hand in the pocket during the sessions in both conditions, ensuring that only tactile stimulation differentiated the two conditions.

### 2.7 Main study: heart rate and physical movement measures

We used the same procedure as in the pilot study. Each session began with the child completing a tutorial interaction on the robot before completing both experimental conditions in randomized order. A 10-point Likert scale questionnaire (in Smiley Face Likert format) was administered by the experimenter after the interactions to assess the child’s self-reported calmness and beliefs about the robot’s ability to provide emotional support. The questionnaire can be found in the Supplementary Appendix in Section 5.

### 2.7.1 Participants and sample size determination

We conducted a power analysis to determine the appropriate sample size for our main study based on data from our 14-day pilot study, as shown in Table 1. The power analysis values produced a pooled standard deviation of approximately 8.1 bpm and an effect size (Cohen’s d) of 0.77. With a significance level of a = 0.05, and a desired power of 80% for a two-tailed test using a paired design, the power analysis indicated a minimum required sample size of 14 participants. To account for potential variability in a larger sample and possible minor data loss, we increased our target by 30%, resulting in our final sample of 18 children in the main study.

**TABLE 1:** Mean heart rates (in bpm) for participants with and without interaction.

Participant	With interaction (bpm)	No interaction (bpm)	Difference (bpm)
A	93.1	99.5	6.4
B	96.4	102.4	6.0
Overall mean	95.0	101.0	6.2
Std. Deviation	7.0	9.0	–

### 2.8 Data processing and statistical methods

As introduced in Section 2.3, we approximated body stillness by quantifying the relative displacement of tracked body joints over time. This approach transformed raw video data into a sensitive behavioral correlate for restlessness, allowing for a continuous movement metric that could be statistically compared against heart rate fluctuations and self-reported measures. For each captured body joint j and image frame f , the movement distance $D_{j,f}$ was calculated using standard Euclidean distance (in image pixels) between consecutive frames, measured as:

$$D_{j,f}=\sqrt{{\left(x_{j,f}-x_{j,f-1}\right)}^{2}+{\left(y_{j,f}-y_{j,f-1}\right)}^{2}}$$ (1)

where $\left(x_{j,f},y_{j,f}\right)$ are the pixel coordinates of joint j in frame f . The videos were recorded at 30 frames per second, such that consecutive measurements were separated by a fixed interval of Δt=1/30s . The lengths of these movement vectors were transformed to SI standard measurement of meters based on the camera’s field of view (84°) and distance to the tracked objects (2 m). Total joint movement was computed as the sum of these distances per second. While pixel-to-distance conversion introduces measurement error, the error was systematic and affected both conditions equally, so it did not bias the comparisons between conditions. Statistical analysis consisted of paired-samples t-tests to assess within-subject differences in both heart rate and movement between conditions. For heart rate analysis, we examined the mean differences in beats per minute between conditions. For movement analysis, we compared average displacement per second between conditions. We also calculated Pearson’s correlation coefficient to explore relationships between heart rate reduction and movement changes, to explore whether physiological calming corresponded with behavioral stabilization. Effect sizes for significant differences were calculated using Cohen’s d. We also examined correlations between physiological responses and self-reported attitudes using Pearson’s correlation coefficient (r) as well as using the Random Forest algorithm. Heart rate (bpm) and physical movement (mean displacement across 15 body points) were correlated with Likert questionnaire responses to assess self-reported calmness and beliefs about robot helpfulness.

## 3 Results

### 3.1 Heart rate

Figure 4 presents the average heart rate measurements for each participant for both experimental conditions. On average, participants experienced a heart rate reduction of 3.37 bpm during the With-Interaction condition compared to the Without-Interaction condition, with a standard deviation of 5.48 bpm. A paired-samples t-test demonstrated a significant difference between conditions ( t(17)=2.60 , p=.019 , 95% CI [0.64,6.09] bpm ), confirming that active tactile interaction with the robot produced consistently lower heart rates than only holding the passive device in the control condition. The effect size (Cohen’s d = 0.32) indicates a small-to-medium effect. Notably, the 3.37 bpm average reduction is comparable to heart rate decreases achieved through established relaxation techniques such as meditation or controlled breathing exercises, which typically require daily extended practice periods of 20 min or more to be effective (Russo et al., 2017; Zaccaro et al., 2018; Perciavalle et al., 2017). The brief interaction with AffectaPocket achieved similar physiological calming effects within two minutes (as tested in the main study for each condition) and without prior training.

**FIGURE 4:**
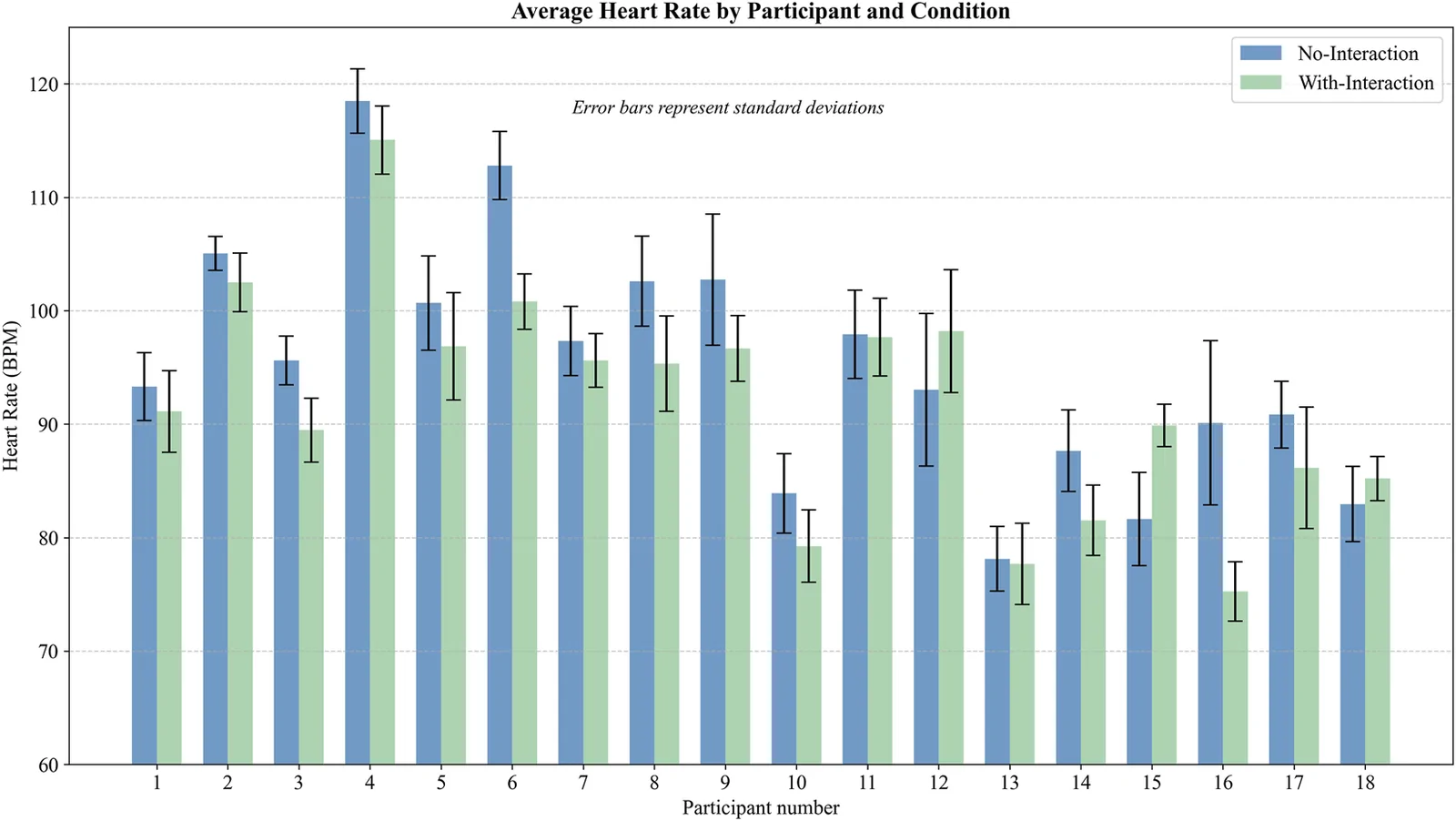
The average difference in heart rate for each of the two conditions per participant; blue bars indicate the Without-Interaction condition and green bars the With-Interaction condition. (Figure reproduced from results in (Frederiksen et al., 2024a)).

An analysis of the onset timing of changes in heart rate during the interaction with the handheld robot revealed a two-phase response pattern in the With-Interaction condition, as outlined in Figure 5. During the Initial Engagement Phase (first 5 s), 44.4% of participants (8 out of 18) showed a clear heart rate pattern where their measurements were initially stable or slightly elevated in the With-Interaction condition. At the first recorded measurement point (time = 0s), the With-Interaction average was 95.0 bpm (SD = 9.58), whereas the Without-Interaction condition was 93.78 bpm (SD = 11.89). A paired-samples t-test showed that this initial difference was not statistically significant (mean difference = 1.22 bpm, 95% CI [ − 2.93, 5.37], t(17) = 0.62, p = 0.543, Cohen’s $d_{z}$ = 0.15). Following the first 5 s, a Settling-In Phase began, with heart rate declining as participants become more focused. This decline seemed most pronounced in the largest cluster of participants (50% of those showing a pattern of declining heart rate), beginning between 3 and 6 s (average 4.5 s), while others showed similar patterns with delayed onset times (median onset at 12 s). The overall lower heart rate in the With-Interaction condition (M = 92.33, SD = 10.43) compared to Without-Interaction condition (M = 95.73, SD = 11.26) appears to be highly impacted by this early settling-in effect, as shown in Figure 5. Focused engagement with the tactile interaction leads to a calmer physiological state that persists throughout the session, as demonstrated by 83.3% of participants (15 out of 18) showing lower average heart rates in the With-Interaction condition.

**FIGURE 5:**
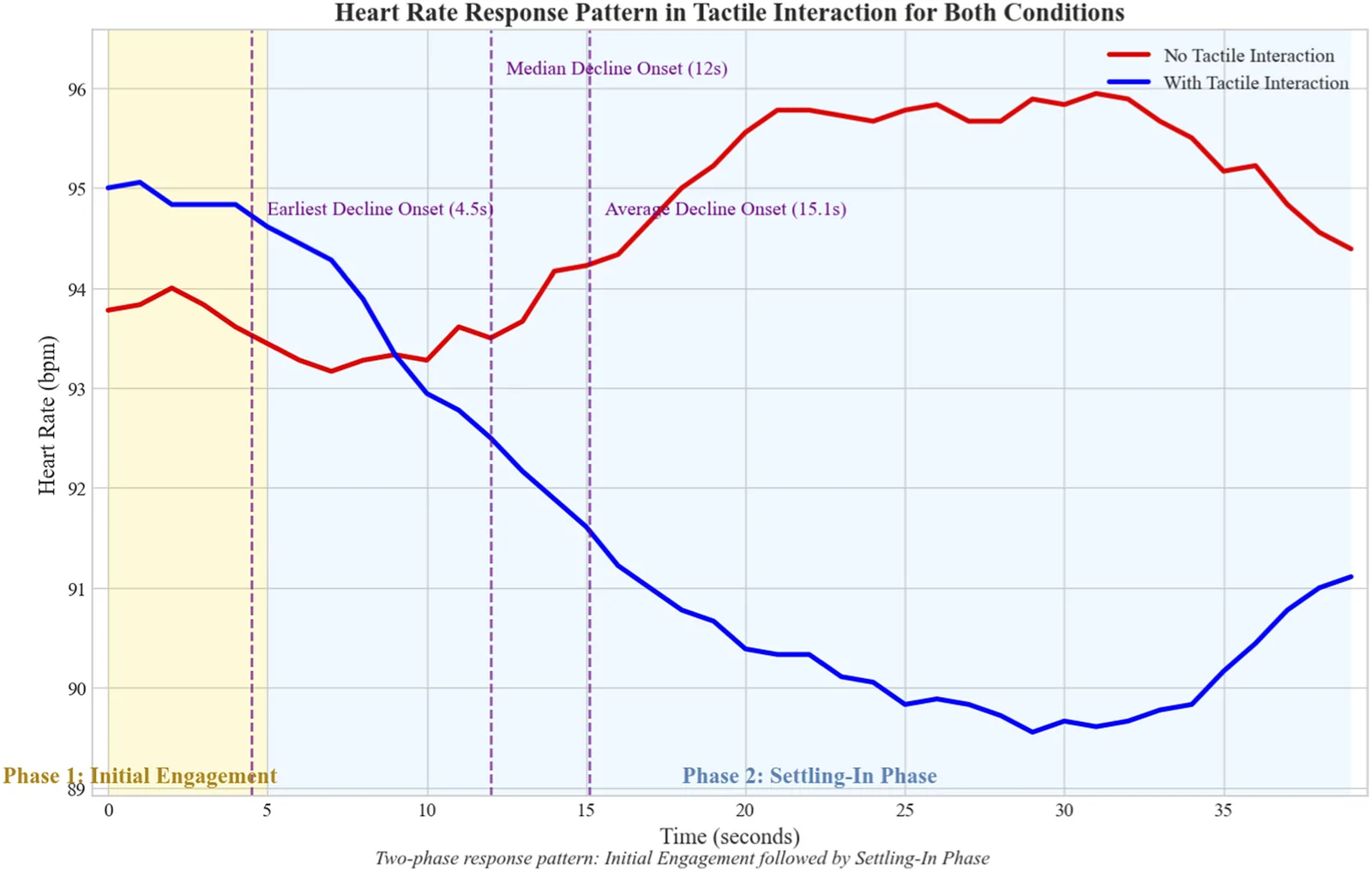
The average difference in initial onset times for heart rate changes for each of the conditions over the initial 40 s of the interaction. The blue line shows the With-Interaction condition and the red line shows the Without-Interaction condition.

### 3.2 Body movement

The child participants in the study showed less overall movement when interacting with the tactile game in the With-Interaction condition (with no explicit difference in freedom given between conditions). Table 2 depicts the average body movement decrease for each condition. Overall movement reduction is evident as most participants (15 out of 18, or 83.3%) showed reduced full body movement during the With-Interaction compared to Without-Interaction, with a significant (paired-samples t-test, t(17) = 3.75, p=.002 , mean difference = 84.0 cm, 95% CI [36.7, 131.3]) average decrease from 223.5 cm (SD = 135.6 cm) to 139.4 cm (SD = 56.9 cm) (37.6% reduction). We also analyzed the magnitude of the movement difference using effect size measures. The Cohen’s d for the paired difference in movement (Without-Interaction vs. With-Interaction) was calculated at d = 0.88, meaning the difference in movement between conditions is not only statistically significant but was substantial in size. The large effect indicates that tactile interaction with the robot had a substantial impact on reducing physical activity levels. Individual variability is reflected in the high standard deviations (135.6 cm for Without-Interaction, 56.9 cm for With-Interaction), showing large individual differences in the individual movement levels and responses to the tactile game intervention, with some children being more active than others during either condition. A few children (3 out of 18) had the opposite effect: they displayed increased body movement in the With-Interaction condition (the largest increase was 31%). Figure 6 shows participant movement over time, depicting summed movement vectors (in meters) throughout the interaction. The plotted time series reveals that the with-tactile-interaction condition generally demonstrated lesser overall movement than the Without-Interaction condition. The consistently lower movement suggests that the tactile game may help to reduce physical restlessness.

**TABLE 2:** Summary of movement data comparing the first 60 s to the full interaction. For each measurement type, the table shows mean movement values for the Without-Interaction and With-Interaction conditions, the mean difference between conditions, and the number of participants showing reduced movement during With-Interaction.

Measurement condition	Without interaction	With interaction	Difference	Participants showing effect
First 60 s				
Full body	873.3	502.3	371.0	15 of 18
Head/Neck/Shoulder joints	230.1	118.0	112.1	16 of 18
Full dataset				
Full body	893.5	557.5	336.0	15 of 18
Head/Neck/Shoulder joints	232.8	128.5	104.3	16 of 18

**FIGURE 6:**
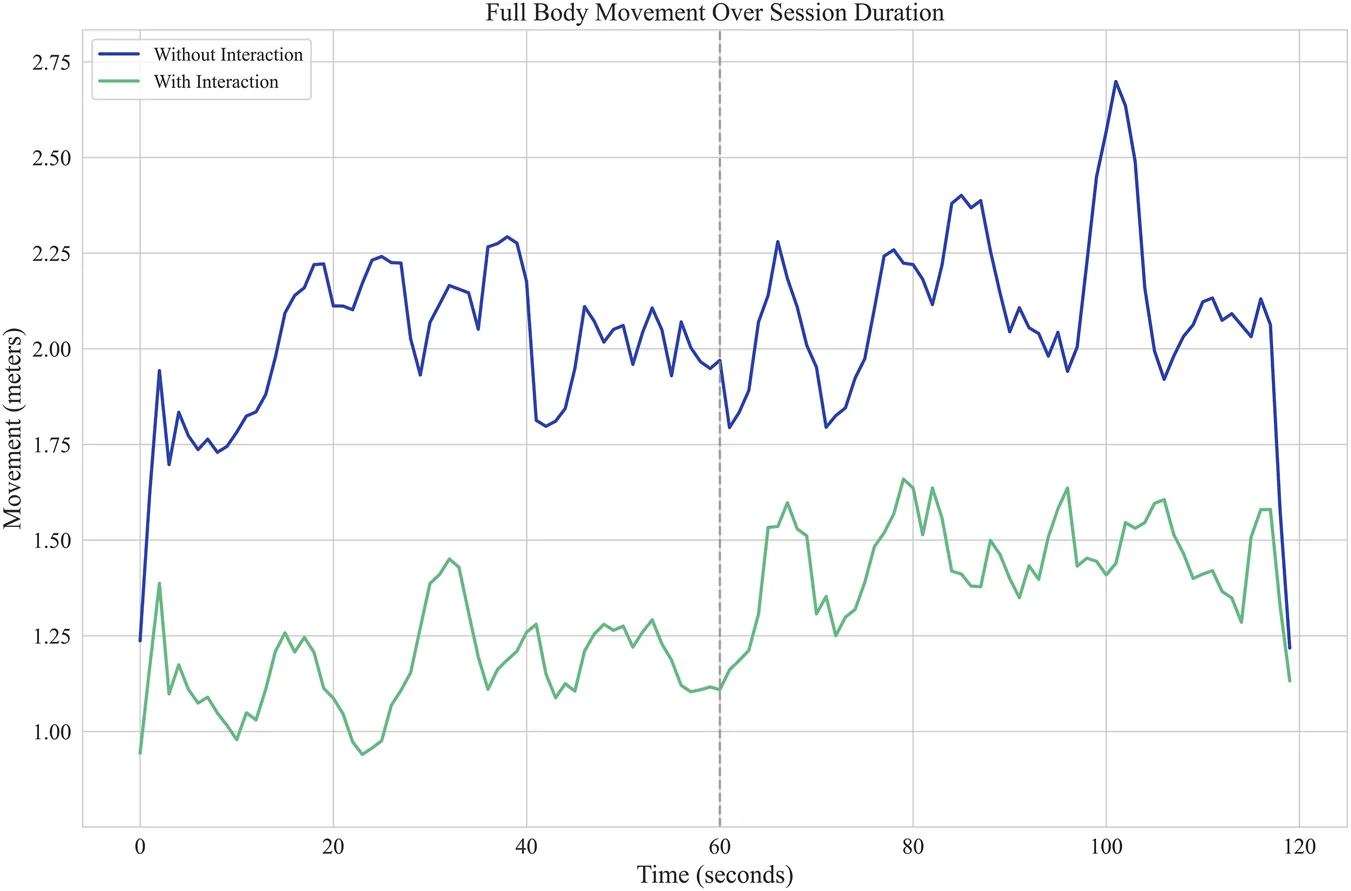
This time series depicts participant-level average body movement vectors including all tracked joints for each condition With-Interaction and Without-Interaction across the two minute study. The graph has been smoothed with rolling average. Lower values indicate less movement.

We also compared movement in a predefined set of head/neck/shoulder joints:

- Eyes (left and right eye), Face (nose) - reflecting head orientation
- Neck - reflecting head and upper-body displacement
- Shoulders (left and right) - reflecting upper-body posture and orientation

When focusing on the predefined head/neck/shoulder joints, the movement difference between conditions was also pronounced. Figure 7 depicts the difference between conditions for both full body movements and head/neck/shoulder joints, highlighting the difference in calculated means between the initial 60 s of the interaction, the final 60 s of the interaction, and the full interaction. The mean movement for these head/neck/shoulder joints is: 58.2 cm (SD = 45.2 cm) in the Without-Interaction condition, and 32.1 cm (SD = 21.2 cm) in the With-Interaction condition. This represents a significant (paired-samples t-test, t(17) = 3.68, p=.002 , mean difference = 26.1 cm, 95% CI [11.1, 41.0], Cohen’s $d_{z}=0.87$ ) 44.8% reduction in movement during the tactile interaction, compared to the 37.6% full body movement reduction, suggesting that the active interaction was associated with especially reduced movement in upper-body and head-position regions.

**FIGURE 7:**
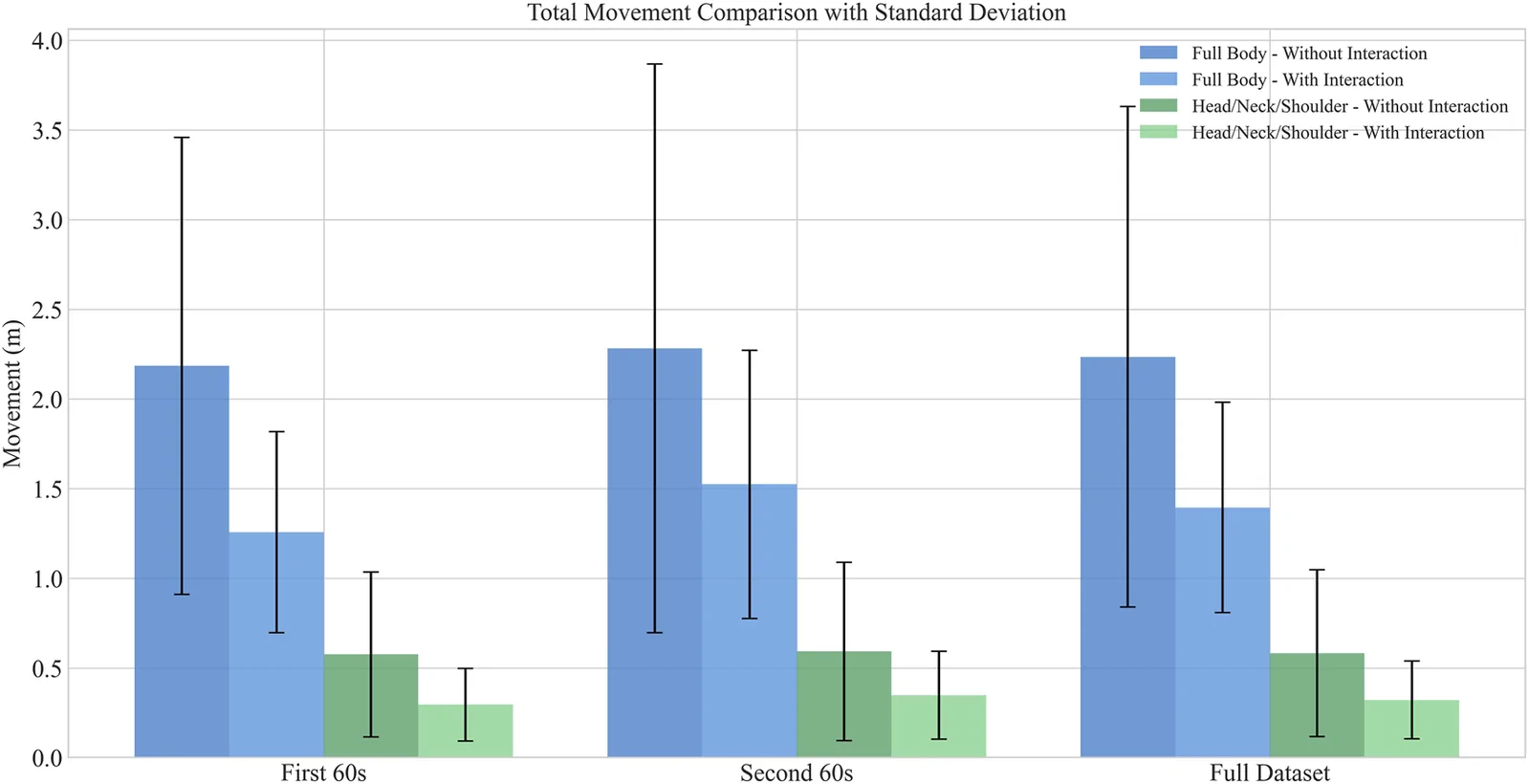
The average total movement and standard deviation for all users per condition. The graph shows full body movements (all tracked joints, and the head/neck/shoulder joints (analyzed in 3.2).

For the remaining isolated body regions, tactile interaction significantly reduced total movement in the head region ( t(17)=3.79 , p=0.002 , 95% CI [6.5, 22.7] cm, Cohen’s $d_{z}=0.89$ ), upper limbs ( t(17)=3.58 , p=0.002 , 95% CI [21.0, 81.1] cm, Cohen’s $d_{z}=0.84$ ), and torso ( t(17)=3.56 , p=0.002 , 95% CI [5.9, 23.0] cm, Cohen’s $d_{z}=0.84$ ).

In the lower limbs, however, the difference was not statistically significant ( t(17)=1.97 , p=0.066 , 95% CI [ − 0.8, 23.4] cm, Cohen’s $d_{z}=0.46$ ). At the individual joint level, most joints in the head, arms, and torso also showed significant decreases (p<0.05) , including the right hip ( t(17)=2.54 , p=0.021 , 95% CI [0.6, 6.4] cm, Cohen’s $d_{z}=0.60$ ), while right knee ( t(17)=0.02 , p=0.985 , 95% CI [ − 11.5, 11.7] cm, Cohen’s $d_{z}=0.00$ ) and left knee ( t(17)=2.07 , p=0.054 , 95% CI [ − 0.07, 7.68] cm, Cohen’s $d_{z}=0.49$ ) did not show significant decreases.

### 3.3 Temporal interaction analysis

As the heart rate data suggest an increased impact within the first half of the interaction, we analyzed the movement data to compare differences in the two-halves of the interaction.

Table 2 presents the movement averages, comparing the initial 60 s against the full interaction period. The results indicate that the reduction in head/neck/shoulder movement was particularly consistent in the initial phase, with 88.9% (16 of 18) of participants showing reduced movement. In terms of magnitude, the movement reduction appeared numerically larger during the first 60 s compared to the second 60 s for both head/neck/shoulder joints (Mean Difference = 28.0 cm vs. 24.2 cm) and full-body movement (Mean Difference = 92.8 cm vs. 75.6 cm). However, paired sample t-tests revealed that these differences between the initial and final phases were not statistically significant for head/neck/shoulder joints ( t(17)=0.68 , p=0.51 , mean difference = 3.7 cm, 95% CI [ − 7.8, 15.1], Cohen’s $d_{z}=0.16$ ) or full-body movement ( t(17)=0.85 , p=0.41 , mean difference = 16.8 cm, 95% CI [ − 24.8, 58.3], Cohen’s $d_{z}=0.20$ ). The lack of statistical significance between phases can be explained by high inter-participant variability (SD = 83.9 cm). While 66.7% of participants (12 of 18) showed the expected stronger effect in the first minute, some outliers exhibited a delayed response, with one participant showing a 184 cm greater reduction in the second phase. This suggests that the movement difference between active tactile interaction and passive holding appeared early for many participants, but individual responses varied substantially, requiring a larger sample size to appear statistically.

### 3.4 Heart rate and self-assessment questionnaire data

Analysis of correlations between the self-reported questionnaire data and heart rate measurements revealed one significant relationship, together with several weak and non-significant trends. All correlations are reported as Pearson’s r with df=16 (n=18) , and change is expressed as the reduction from the Without-Interaction to the With-Interaction condition, such that negative correlations indicate smaller reductions among children with higher ratings.

Self-reported calmness was significantly correlated with initial heart rate, in both the Without-Interaction ( r(16)=0.59 , 95% CI [0.17, 0.83], p=0.010 ) and With-Interaction ( r(16)=0.53 , 95% CI [0.09, 0.80], p=0.023 ) conditions: children who described themselves as calmer began the session with higher heart rates, a counter-intuitive direction. Self-assessment therefore appears to relate to where children started rather than to how much they changed, which frames the remaining results. Consistent with this, children’s belief in the robot’s ability to help them in a stressful situation showed only a small, non-significant negative correlation with heart rate change during interaction ( r(16)=−0.34 , 95% CI [ − 0.69, 0.15], p=0.171 ), as did belief in the robot’s effectiveness for offering support with movement differences ( r(16)=−0.23 , 95% CI [ − 0.63, 0.26], p=0.353 ) and self-reported calmness with heart rate change ( r(16)=−0.21 , 95% CI [ − 0.62, 0.28], p=0.400 ). Participants with high confidence in the robot ( ≥ 8, n=11 ) tended to begin the session with lower heart rates (93.1 bpm, SD = 12.83, at Time = 0) than those with medium to low confidence ( < 8, n=7 ) who started with higher heart rates (94.9 bpm, SD = 11.14, at Time = 0), though this relationship was not significant ( r(16)=−0.20 , 95% CI [ − 0.61, 0.30], p=0.435 ). Given that nine correlations were examined, the calmness–initial heart rate relationship would not survive correction for multiple comparisons ( $p_{\text{adj}}=0.09$ , Bonferroni) and should be treated as exploratory. Overall, these results suggest that preexisting attitudes about robots may be more closely associated with children’s baseline physiological state than with the magnitude of their response to interaction, and require further studies to explore this relationship.

### 3.5 Heart rate and physical movement

The analysis of the relationship between physical movement and heart rate across conditions revealed no significant correlations when examining simultaneous movement and heart rate patterns. However, when investigating time-delayed effects (first 30 s of movement and last 20 s of measured average heart rate), we found a moderate negative correlation (r = −0.37, 95% CI [-0.71, 0.12], p = 0.131), indicating that higher initial movement might predict lower subsequent heart rate. Because this relationship was not statistically significant, the present data do not support strong conclusions about coupling between movement change and heart-rate change.

### 3.6 Random forest analysis

To explore potential non-linear relationships and interactions between the subjective variables of the post-experiment questionnaire and the heart rate and movement measurements, we analyzed the data using a Random Forest algorithm with resampling across 1,000 iterations. This analysis revealed that children’s belief in the robot’s ability to help was consistently the most important predictor for both physiological measures, showing moderate importance for heart rate changes (importance score: 0.34) and movement differences (importance score: 0.23). However, none of these predictors reached statistical significance. The limited sample size (n = 18) of our study could explain the lack of statistical power in these results.

### 3.7 Hypothesis testing

Our findings provide the following evidence relative to our study hypotheses:

- H1: Children’s total physical movement will be significantly reduced during active engagement with the robot’s tactile game compared with the Without-Interaction condition.
  H1 is supported. The overall reduction in movement for the With-Interaction condition was statistically significant (t(17) = 3.75, p = 0.002, mean difference = 0.84 m, 95% CI [0.37, 1.31]), with 83.3% of participants (15 out of 18) showing reduced full body movement during the session for this condition. On average, movement decreased from 2.23 (SD = 1.36) meters to 1.39 (SD = 0.57) meters, representing a 37.6% reduction. The Cohen’s d value of 0.88 indicate that this effect is not only statistically significant but also substantial in magnitude.
- H2: The children’s upper body parts (head, eyes, and upper body) will show greater movement reduction than other body areas during tactile interaction with the pocket-sized robot.
  H2 is supported. When focusing only on head/neck/shoulder joints, the effect of tactile interaction was more pronounced. The mean movement for these joints showed a 44.8% reduction during the With-Interaction condition (from 0.58 M to 0.32 M), compared to the 37.6% reduction in overall body movement (t(17) = 3.68, p = 0.002, mean difference = 0.26 m, 95% CI [0.11, 0.41], Cohen’s $d_{z}$ = 0.87). This suggests that tactile interaction with the robot particularly affects the movement of the upper body parts.
- H3: The magnitude of the children’s heart rate reduction will positively correlate with the degree of movement reduction.
  H3 is not supported. Our analysis revealed no significant correlations when examining simultaneous movement and heart rate patterns. When investigating time-delayed effects (first 30 s of movement and last 20 s of measured average heart rate), we found a moderate negative correlation (r(16) = −0.37, 95% CI [ − 0.71, 0.12], p = 0.131), suggesting that higher initial movement might predict lower subsequent heart rate. However, this correlation did not reach statistical significance, and the direction was opposite to our hypothesis. This indicates that heart rate and movement reductions, while both significant individually, likely represent separate regulatory mechanisms rather than an integrated response.
- H4: The magnitude of the condition difference will be time-dependent, resulting in a more pronounced difference between the With-Interaction and Without-Interaction conditions during the final phase (the last 60 s) of the session compared to the initial phase.
  H4 is not supported. The movement difference between conditions was numerically larger during the first 60 s than during the second 60 s, but this temporal difference was not statistically significant (full body: t(17) = 0.85, p = 0.41, 95% CI [ − 24.8, 58.3] cm; head/neck/shoulder: t(17) = 0.68, p = 0.51, 95% CI [ − 7.8, 15.1] cm). The reduction in head/neck/shoulder movement was particularly consistent in the initial phase, with 88.9% of participants showing reduced movement in these regions. These findings suggest that the movement difference between active tactile interaction and passive holding appeared early for many participants, but individual responses varied substantially.

In summary, our results support two of our hypotheses (H1 and H2) that the active tactile rhythm-matching condition was associated with lower heart rate and reduced movement compared with passive device holding, and that it was more pronounced in the upper body parts. Our findings do not support the hypotheses regarding the correlation between movement and impact on heart rate (H3) or the time-dependent nature of changes in heart rate and movement (H4).

## 4 Discussion

### 4.1 Reduced movement during active tactile interaction

The results of our study suggest that children moved less during active tactile rhythm-matching interaction than while passively holding the inactive device. Overall activity was reduced by about 37.6% when tactile stimulation was present, and the largest reductions appeared in the head, neck, and shoulder regions. Taken together, these findings indicate that tactile interaction with the robot was associated with a measurable reduction in physical movement during the brief interaction period.

One possible interpretation is that the tactile rhythm-matching task provided a structured point of engagement that helped reduce extraneous movement. In this sense, the device may have functioned as a simple sensory-motor anchor: the child had something concrete to hold, feel, and respond to, which may have organized their movement during the interaction. However, the functional meaning of reduced movement should be interpreted cautiously. Less movement is not necessarily equivalent to better attention, better self-regulation, or improved functioning. Fidgeting and purposeful movement can sometimes support arousal regulation, stress management, or task engagement, and research on fidget devices has produced mixed findings. Accordingly, the present results should be interpreted as reduced movement during active tactile task engagement, rather than as direct evidence that the device improved attention or self-regulation abilities.

This interpretation is consistent with, but does not directly test, sensory integration perspectives suggesting that tactile input can help some children become more settled during activity (Ayres et al., 2005). Related work with children with attention difficulties has also reported that targeted tactile interventions can reduce hyperkinetic behavior and support self-regulation (VandenBerg, 2001; Pfeiffer et al., 2008). The present study extends this line of work in a more limited and acute context: typically developing children interacted briefly with a pocket-sized tactile robot, and during that interaction they showed reduced movement compared with passive device holding. Future studies are needed to determine whether this reduction reflects calmer behavior, task-related postural stabilization, novelty, attentional engagement, tactile feedback, or a combination of these factors.

The present findings therefore support the potential of pocket-sized tactile devices as momentary interaction tools, but not yet as comprehensive interventions. The observed reductions in movement and heart rate were short-term physiological and behavioral changes measured within a controlled interaction setting. Longitudinal and task-matched studies are needed to determine whether these acute changes translate into meaningful functional benefits across contexts, such as improved tolerance of stressful situations, better task persistence, or reduced subjective distress.

### 4.2 Heart rate and movement disparities

The active tactile rhythm-matching condition was associated with both lower heart rate and lower measured movement. However, the lack of a significant correlation between movement reduction and heart-rate change suggests that these two outcomes should not be interpreted as a single coupled response. Children who showed the largest reductions in movement were not necessarily the same children who showed the largest reductions in heart rate. This distinction is important because it prevents a simple explanation in which lower heart rate is assumed to be only a consequence of reduced physical activity, or in which reduced movement is assumed to directly reflect reduced physiological arousal.

Instead, the findings may indicate that the interaction affected physiological and motor activity in partially separate ways. The rhythm-matching interaction required children to hold the device, attend to tactile cues, and produce timed button presses, while also remaining within the camera frame. Any of these features could have shaped movement patterns independently of heart-rate change. Similarly, the lower heart rate observed during active interaction could reflect several possible mechanisms, including rhythmic engagement, tactile stimulation, novelty, task structure, distraction from the experimental setting, or a more settled posture during interaction. The present study was not designed to isolate these mechanisms, but the divergence between heart-rate and movement changes suggests that both outcomes should be considered separately in future work.

This interpretation also has implications for how the device should be evaluated in future potential high-arousal contexts. Elevated arousal and physical restlessness often co-occur in stressful situations, but they are not interchangeable outcomes. A child may become physically still without becoming physiologically calmer, or may show reduced physiological arousal while continuing to move. Therefore, the value of tactile interaction should not be judged from movement reduction alone. Future studies should examine heart rate, movement, subjective state, and task performance together, especially in contexts where the functional meaning of movement is clearer, such as waiting, sustained attention, public-speaking anticipation, or other stress-inducing situations.

Rather than treating these possible mechanisms as competing explanations, future work can use them to better understand what makes tactile robotic interaction effective. The present findings suggest that the interaction may have value precisely because it combines several features that are difficult to separate in everyday use: tactile feedback, rhythmic engagement, handheld motor activity, and the playful novelty of a small robot. This combination may be especially relevant for children, for whom arousal regulation often develops through scaffolded, concrete activities and sensory-motor experiences rather than through internal strategies alone (Florez, 2011; Morawska et al., 2019; Xu et al., 2025). From this perspective, the lack of a direct correlation between heart-rate change and movement reduction may be informative: the device may support a broader state of engagement in which physiological arousal or physical restlessness can shift without necessarily changing in synchronization. Future studies can build on this by examining which elements of the interaction are most important, and in which contexts the combined tactile, rhythmic, and robotic qualities are most beneficial.

### 4.3 Limitations and methodological considerations

The present study evaluated the tactile robot as a complete interaction experience, combining tactile feedback, rhythm matching, handheld motor activity, and structured engagement. This reflects the way the device is intended to be used by children, where these elements operate together rather than as isolated components. A valuable direction for future work is to examine the relative contribution of these components by varying features such as tactile feedback, rhythm matching, or active button pressing across comparison conditions. Such studies would help identify which aspects of the interaction are most influential, while the present findings provide an initial evaluation of the combined tactile robotic interaction.

The authors also acknowledge the following limitations of the study: First, the small sample size (n = 18) limits statistical power and generalizability of the findings. Second, the experimental setting required children to stand on a marked spot to ensure that they were captured by the camera, which introduced an artificial constraint on natural movement. This experimental constraint of being stationary in a specific location could affect children’s natural movement patterns, possibly increasing restlessness in the Without-Interaction condition as children attempted to comply with instructions to remain stationary. Future studies should include a more diverse sample in less constrained environments, allowing for more naturalistic movement assessment, to determine if the effects observed here generalize to standard school contexts in a larger sample of participating children.

### 4.4 Future directions: stressful and attention-demanding contexts

The findings from this study suggest that handheld pocket-sized tactile devices may be worth investigating in contexts where children experience heightened arousal, restlessness, or stress. AffectaPocket was associated with lower physiological arousal, measured as an average heart-rate decrease of 3.37 bpm (95% CI [0.64, 6.09], t(17) = 2.60, p = 0.019, d = 0.32), and lower measured movement, with overall movement reduced by 37.6% (95% CI [36.7, 131.3] cm, t(17) = 3.75, p = 0.002, dz = 0.88), during brief tactile interaction. These outcomes are relevant because stressful and anxiety-related states in children often involve both internal physiological symptoms and external behavioral signs, including somatic discomfort, elevated arousal, and visible restlessness (Association, 2022; Beesdo et al., 2009; Ginsburg et al., 2006). The present findings therefore do not establish anxiety reduction, but they do show that the device influenced markers that are important to consider in future anxiety- and stress-related studies.

A central potential of this approach is that the interaction is concrete, immediate, and easy for children to initiate. Traditional therapeutic approaches, including cognitive-behavioral strategies, can be highly effective, but they often require instruction, repeated practice, and support before children can use them independently in difficult moments (Kendall and Hedtke, 2006; Oar et al., 2017; Rapee et al., 2009). In contrast, a handheld tactile robot may offer a simple embodied activity that children can engage with quickly and with little preparation. This does not position the device as a replacement for established interventions, but rather as a possible complementary tool for moments when children may benefit from accessible, in-the-moment support (Creswell et al., 2014; Velting et al., 2004).

This direction is particularly relevant given the prevalence of childhood anxiety and the limited availability of timely mental health resources for many children and families (Bitsko et al., 2022; Widiger and Hines, 2022). However, the present study was conducted with typically developing children in a familiar, low-stress classroom environment, and the results should not be generalized directly to clinical populations. Instead, the study provides a proof-of-concept showing that brief tactile robotic interaction can influence physiological and behavioral markers that are relevant for future work in more demanding contexts.

Future studies should therefore examine AffectaPocket during situations in which stress, anxiety, or attentional demands are more directly present or in validated stress-induction paradigms. Such work would help determine whether the acute reductions in heart rate and movement observed here translate into meaningful benefits, such as improved task persistence, reduced subjective distress, or better tolerance of stressful situations.

## Data Availability

The raw data supporting the conclusions of this article will be made available by the authors, without undue reservation.
