# Supplementary material for: Tactile regulation with pocket-sized robots: reducing movement and heart rate in children through hand-held interaction
Source: Front Robot AI. 2026 Sep 23;13:1810778. doi: 10.3389/frobt.2026.1810778 (PMC13642002; doi:10.3389/frobt.2026.1810778)

Introductory Question If you were to say how calm you become when playing the game, where 1 is "very little" and 10 is "a lot"?

- (Scale: 1 = Very little to 10 = A lot)

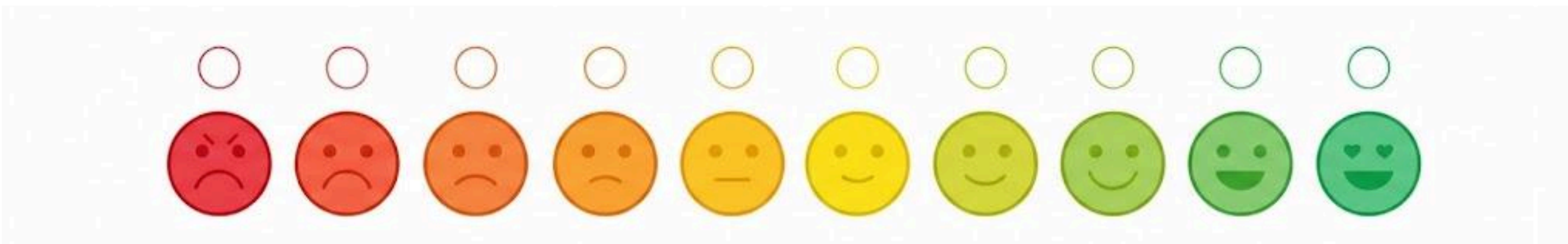

Q1: When the robot gives you points, how much does it make you want to play with it?

- (Scale: 1 = Very little to 10 = Very much)

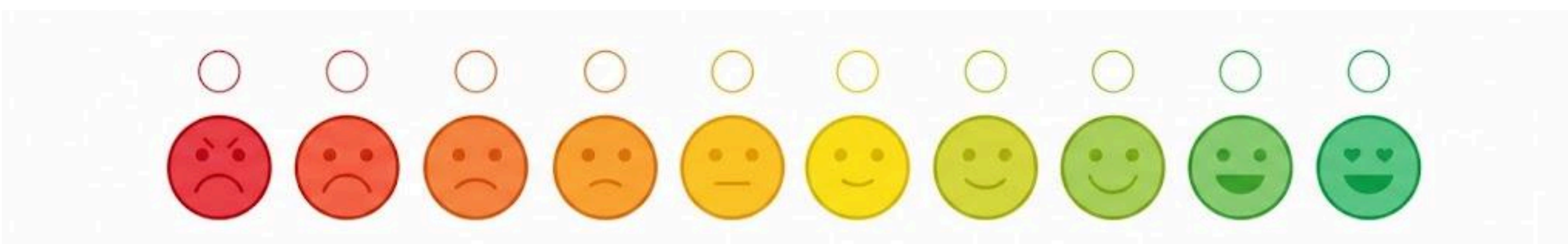

Q2: When you make the robot happy by playing with it, how much does it make you want to play with it?

- (Scale: 1 = Very little to 10 = Very much)

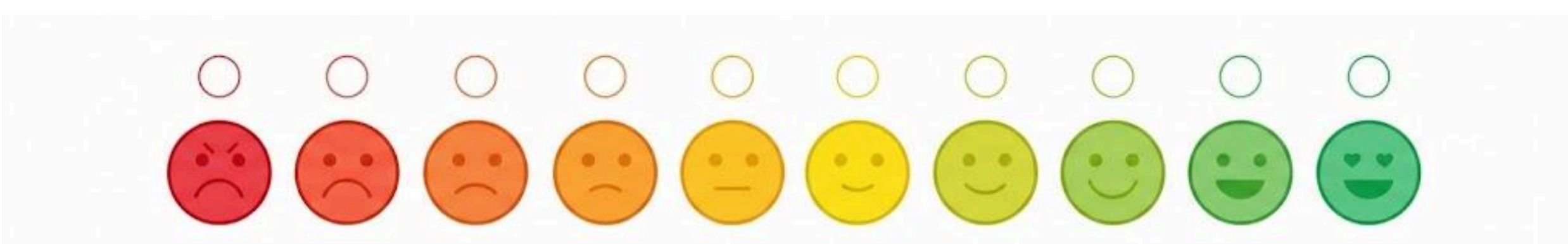

Q3: Do you like it best when the robot becomes happy, or when it gives you points?

- (Open ended/Multiple choice)

Q4: How much do you think you could help the robot if it were sad?

- (Scale: 1 = Very little to 10 = A lot)

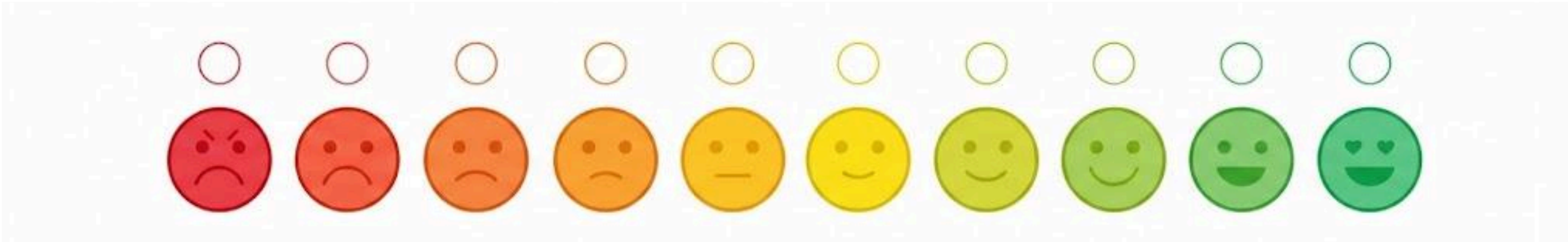

Q5: How much do you think the robot could help you if you were sad?

- (Scale: 1 = Very little to 10 = A lot)

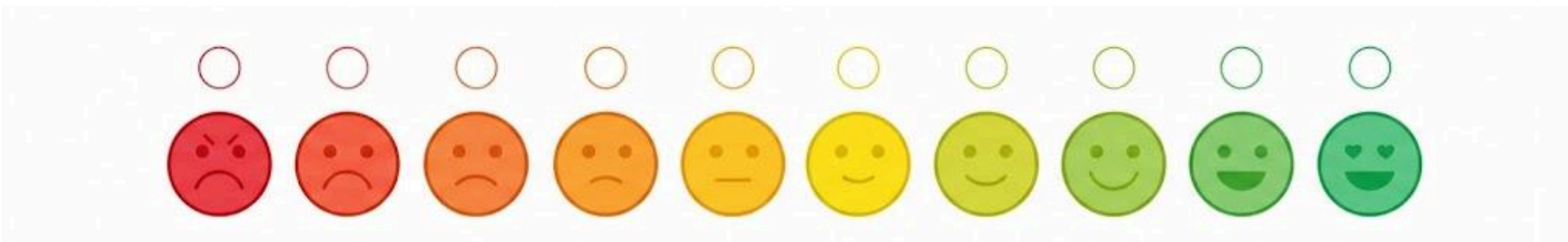

Q6: How much do you think you could help the robot if it were afraid?

- (Scale: 1 = Very little to 10 = A lot)

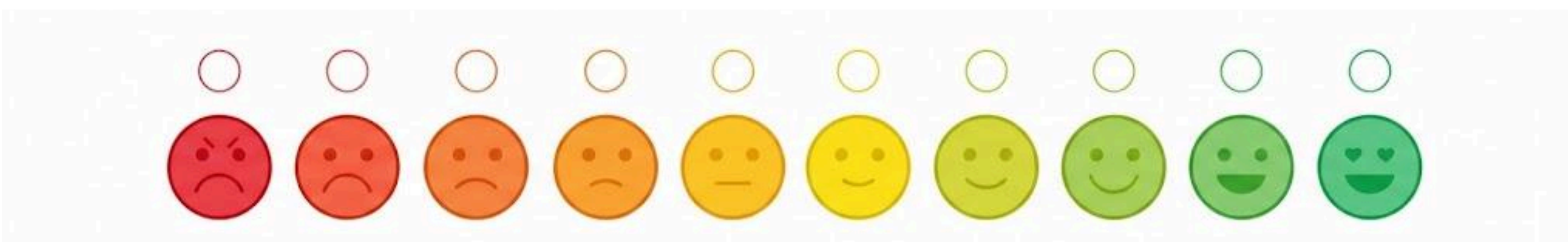

Q7: How much do you think the robot could help you if you were afraid?

- (Scale: 1 = Very little to 10 = A lot)

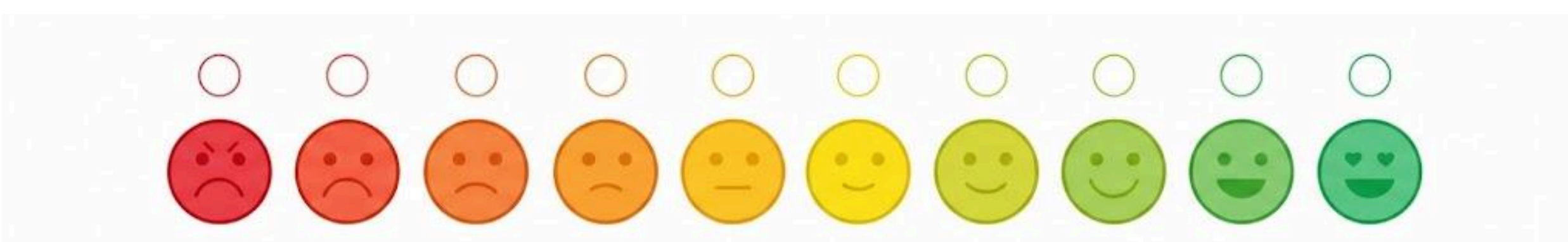

Q8: How much do you like the robot? *(Please rate your impressions of the likability of the robot on the following scale)*

- (Scale: 1 = Not friendly to 10 = Very friendly)

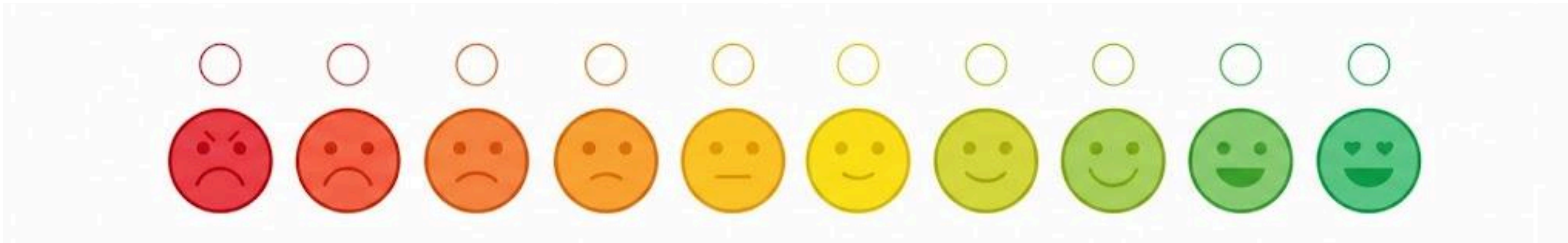

Q9: How friendly do you think the robot is? *(Please rate your impressions of the likability of the robot on the following scale)*

- (Scale: 1 = Not friendly to 10 = Very friendly)

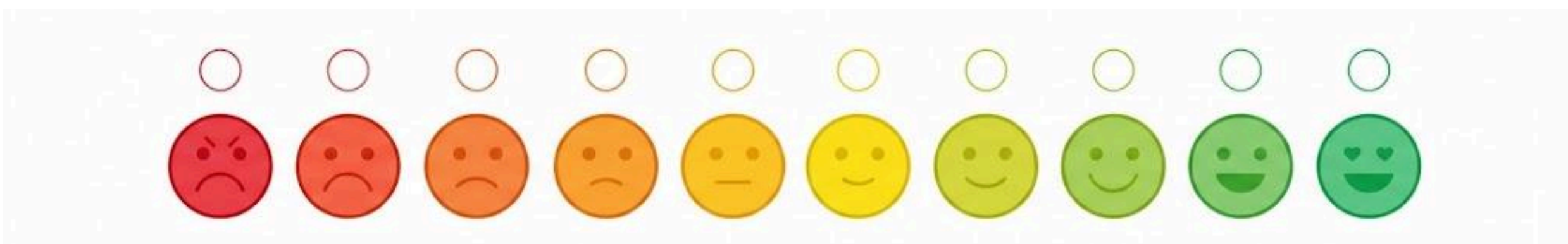

Supplement: Supplementary file 1 [file DataSheet1.pdf]
